# Evaluating the Relationships between Riparian Land Cover Characteristics and Biological Integrity of Streams Using Random Forest Algorithms

**DOI:** 10.3390/ijerph18063182

**Published:** 2021-03-19

**Authors:** Se-Rin Park, Suyeon Kim, Sang-Woo Lee

**Affiliations:** 1 Graduate Program, Department of Forestry and Landscape Architecture, Konkuk University, Gwangjin-Gu, Seoul 05029, Korea; serin87@konkuk.ac.kr (S.-R.P.); mdln94@konkuk.ac.kr (S.K.); 2 Department of Forestry and Landscape Architecture, Konkuk University, Gwangjin-Gu, Seoul 05029, Korea

**Keywords:** riparian land cover, spatial pattern, biological indicator, random forest, threshold analysis, South Korea

## Abstract

The relationships between land cover characteristics in riparian areas and the biological integrity of rivers and streams are critical in riparian area management decision-making. This study aims to evaluate such relationships using the Trophic Diatom Index (TDI), Benthic Macroinvertebrate Index (BMI), Fish Assessment Index (FAI), and random forest regression, which can capture nonlinear and complex relationships with limited training datasets. Our results indicate that the proportions of land cover types in riparian areas, including urban, agricultural, and forested areas, have greater impacts on the biological communities in streams than those offered by land cover spatial patterns. The proportion of forests in riparian areas has the greatest influence on the biological integrity of streams. Partial dependence plots indicate that the biological integrity of streams gradually improves until the proportion of riparian forest areas reach about 60%; it rapidly decreases until riparian urban areas reach 25%, and declines significantly when the riparian agricultural area ranges from 20% to 40%. Overall, this study highlights the importance of riparian forests in the planning, restoration, and management of streams, and suggests that partial dependence plots may serve to provide insightful quantitative criteria for defining specific objectives that managers and decision-makers can use to improve stream conditions.

## 1. Introduction

Land development in watershed and riparian areas can significantly alter hydrology, hydraulics, pollution loading, and the transport mechanisms of pollutants, which substantially contribute to poor stream water quality and the degradation of stream ecosystems [1,2]. As extreme weather events, including floods, droughts, and storms, are becoming more frequent and intense, dealing with pollutants from catchment runoff processes (i.e., stormwater runoff) has become a major challenge for policymakers and environmental managers in terms of sustaining stream water quality and aquatic ecosystems [3,4]. In particular, changes in land cover types and spatial patterns are one of the key influencing factors that alter hydrological systems, leading to changes in stormwater runoff characteristics [5,6]. Therefore, it is critical to implement effective watershed management strategies to mitigate the adverse impacts of land cover types and their spatial patterns on the biological communities of streams [7].

Riparian buffers can manage stormwater by mitigating surface runoff processes, such as decreasing flow velocity and increasing residence time [8,9]. It is critical to mitigate surface runoff for efficient infiltration, absorption, evaporation, and interception in riparian buffer zones, which can affect the capacity to control floods, trap sediments, and filter out pollutants and nutrient loading. In addition, riparian zones provide several ecological functions, such as enhancing biodiversity, microclimate regulation, and increasing recreational opportunities [10,11,12]. However, human activities and intensified land use cause fragmentation, loss, and degradation of riparian vegetation, which can negatively affect the biological integrity of streams [13]. The high proportion of anthropogenic land cover types and the degraded spatial pattern of riparian vegetation are responsible for the poor water quality and biological conditions in streams [14,15]. Recently, Yirigui et al. [13] reported that biological conditions in streams deteriorate as the patch size and core area decrease and edge areas increase in riparian forests. Shen et al. [16] identified that forest edge density has a positive impact on stream water quality in eastern Canada. However, several studies on the relationship between watershed spatial patterns and stream conditions have reported that the effects of land cover patterns on stream water quality and biological conditions depend on the spatial scale, metrics used, and land cover types [16,17,18]. Therefore, scientific research that quantifies the relationships between land cover characteristics of riparian buffer zones and biological indicators of streams should be performed [19].

Different statistical methods have been used to study the relationships between land cover characteristics and stream ecosystems, including multiple regression, regression trees, and redundancy analysis. Such conventional statistical methods assume the normality and spatial independence of the observed datasets, as well as linearity and non-multicollinearity between dependent and independent variables [20,21]. In most cases, however, these assumptions are difficult to satisfy in stream monitoring datasets, as land use types, landscape characteristics, water quality, and the biological communities of stream ecosystems are not spatially discrete or independent. Therefore, many studies have adopted machine learning approaches (see, e.g., [22,23,24]), as they do not require such assumptions [25,26]. Among many machine learning approaches, random forest regression has been shown in the literature to be effective and easily interpretable (for example, [20,27]). The random forest approach uses bootstrap aggregation of regression trees and provides better results than other machine learning techniques [25]. Specifically, the random forest approach can handle nonlinear and complex relationships and can determine variable importance with high predictive accuracy [20,26]. For example, Ouedraogo et al. [20] applied the random forest methodology to predict groundwater nitrate contamination, while Giri et al. [27] also used the random forest algorithm to evaluate the relationship between land use intensity and aquatic ecosystems.

As the relationships between landscape indicators and aquatic ecosystems are nonlinear and complex [28], the response of biological communities to riparian land cover may have important change points, where the ecosystem status can be abruptly changed under a small driver [29,30]. Detecting the thresholds of aquatic ecosystems for land cover characteristics in watershed and riparian areas can provide objective scientific criteria for managers and policymakers involved in water pollution control and land use planning [27]. Although several studies have reported that relationships between aquatic ecosystems and land cover indicators are nonlinear and exhibit change points, there have been limited studies that have investigated the change point of the effects of riparian land cover and their spatial patterns on aquatic ecosystems [30,31,32].

This study aims to improve the understanding of the effects of riparian land cover on the biological integrity of streams using machine learning algorithms to handle the associated complex and nonlinear datasets. The overall objectives of this study are as follows: (a) to investigate the relative importance of land cover characteristics in riparian buffer zones on the biological indicators of streams, and (b) to analyze the critical change points and visualize the average effects of riparian land cover proportions and their spatial patterns on biological indicators. The results of this study provide essential insights for establishing management strategies and restoration plans in riparian buffer zones, in order to enhance ecological functions and mitigate the negative effects of stormwater runoff. Additionally, identifying the threshold responses of aquatic ecosystems to riparian land cover can provide objective quantitative criteria for land use zoning regulations and restoration programs.

## 2. Materials and Methods

### 2.1. Study Area

**Figure 1:**
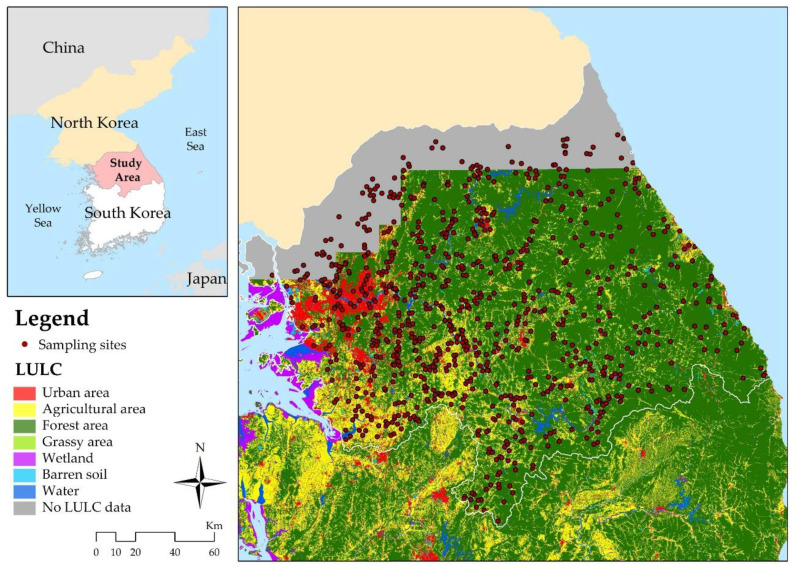
Han River basin, sampling sites, and land use/land cover (LULC). Areas in the north are the border areas with no LULC data.

The Han River is one of four major rivers in South Korea. Its catchment area covers approximately a quarter of the country’s surface (709 km in length and 25,953 km^2^ in area). The Han River is located between 36°30′ and 38°55′ N latitudes and between 126°24′ and 129°02′ E longitudes. The Han River system covers the middle region of the Korean Peninsula and flows from east to west (Figure 1). The climate in the basin is hot and humid during summer, and cold and dry during winter. Average annual (total) precipitation is 1348 mm, and the average annual precipitation during the dry season is 193 mm. As there are distinctive climate characteristics between seasons, river flows vary greatly with the seasons [33]. In the study area, the average annual temperatures range between 12.5 and 13.6 °C, depending upon the region, while the average monthly temperatures vary from −2.5 °C in January to 25.4 °C in August [34]. 

The Ministry of Environment (MOE) of Korea has hierarchically divided watersheds to manage the water environment across the entire country, including the national watershed management regions (NWMRs), base watershed management regions (BWMRs), and sub-watershed management areas (SWMAs). There are six NWMRs, 29 BWMRs, and 265 SWMAs in the Han River basin. The Han River basin is the largest basin in South Korea, which includes Seoul, the capital of South Korea, and the land development pressure and population growth are concentrated, with approximately 53% of the entire population living in the basin. Accordingly, maintaining the water quality and aquatic ecosystem functionality in the Han River basin are essential, as it is a primary water source for human development.

### 2.2. Monitoring Program and Biological Indicators

Under the National Aquatic Ecological Monitoring Program (NAEMP), the Korean MOE monitors rivers and streams using different indicators to evaluate habitat condition, biological community integrity (e.g., the floral community), and other biochemical conditions across the entire nation twice a year. Aquatic organisms, as key indicators of overall basin integrity, can be used to assess the long-term effects of anthropogenic disturbances on streams and overcome the limitations of applying chemical parameters [35]; therefore, the NAEMP monitors the biological status of freshwater ecosystems using three specific groups of aquatic organisms: diatoms, benthic macroinvertebrates, and fish. In the Han River basin, the dominant species in each group were *Achnanthes convergens*, *Baetis fuscatus*, and *Zacco platypus*, respectively. The NAEMP has adopted the following three indicators to evaluate the status of streams (Table 1): the Trophic Diatom Index (TDI) by Kelly and Whitton for diatom communities [36]; the Benthic Macroinvertebrate Index (BMI), developed by the NAEMP for benthic macroinvertebrate communities, and the Fish Assessment Index (FAI), originally proposed by Karr and developed by the NAEMP for fish assemblages [37]. In the present study, these biological indicators were adopted to quantify the impact of riparian land cover and spatial patterns on the status of stream biological communities as indicators of fluvial ecosystem integrity. The biological indicators range from 0 (very poor) to 100 (very good), this scale serving to gauge the relative biological status in streams. More detailed information on these indicators can be found in [35]. For this study, we used biological indicator datasets collected from 2016 to 2018 at 907 monitoring sites. Of these 907 sites, data from only 770 were used in our analyses. The remaining 137 sites were omitted due to gaps in stream monitoring data and an excess of variables and outliers in the land use/land cover (LULC) records.

### 2.3. Land Cover Characteristics of Riparian Buffer Zones

We selected the proportions of land cover type (% land cover) and their spatial patterns to measure the land cover characteristics of the riparian area. To acquire % land cover and spatial patterns in riparian buffer zones, we used a land use/land cover (LULC) dataset obtained from the Korean MOE. The LULC map of the riparian buffer zones was converted into raster data (10 × 10 m), and the land cover pattern metrics were calculated using the FRAGSTATS (version 4.2, The University of Massachusetts, Amherst, MA, USA) software [39]. The original LULC map was classified into seven major categories and 23 subcategories: (a) urban areas including residential, industrial, commercial, and roads; (b) agricultural areas including paddy fields, farms and orchards; (c) forest areas; (d) grassy areas; (e) wetlands; (f) barren soil; and (g) water. We reclassified the original LULC map into the following three categories: (a) urban areas; (b) agricultural areas; and (c) forested and grassy areas. The proportions of each land cover category in the watershed are calculated for urban areas (12.0%), agricultural areas (16.6%), forest areas (51.5%), grassy areas (10.9%), wetlands (1.8%), barren soils (3.9%), and water (3.3%).

As the proposed buffer width varied considerably in previous studies [15,16,19], specifying the scale of riparian buffer zones was challenging. The Korean MOE has designated buffer zones of 1 km width to preserve riparian areas and preserve water quality; therefore, we adopted this riparian criterion to define the buffer width of each stream. Each 1 km buffer zone was created and overlaid on the LULC raster data (10 m resolution). Then, all LULC raster data within the buffer zones were clipped and stored as separate grid files to compute the land cover pattern metrics.

Multiple metrics should be considered to describe the different spatial characteristics of landscapes [40,41]. We selected 12 metrics at the class level, including the large patch index (LPI), percentage of landscape (PLAND), patch density (PD), and edge density (ED) of the individual land cover types (urban, agricultural, and forest areas; see Table 2). The spatial pattern metrics selected for this study were those most often used in previous studies as land cover patterns to explain stream conditions (see, e.g., [6,16,17]).

### 2.4. Statistical Approach

As indicated in the previous introduction, machine learning algorithms, such as artificial neural networks (ANNs), support vector machines (SVMs), and random forests are preferred, as they go beyond traditional statistical methods, owing to their ability to handle non-parametric datasets and nonlinear relationships [22,24,42]. In this study, we adopted the random forest algorithm, which is one of the most powerful machine learning methods, to explore the nonlinear and complex interactions among the land cover characteristics of riparian buffer zones and the biological communities of streams [25]. Random forest algorithms reduce prediction error rates and result in more accurate estimates by creating multiple trees [17,20,26]. As a random forest analysis assesses the effects of all explanatory variables and ranks the importance of these variables, it is possible to detect complicated interactions among variables. Although it is difficult to interpret the overall effect of variables, random forest algorithms have been successfully applied in various research fields [17,26]. Researchers have utilized random forest algorithms to apply partial dependence plots to interpret and visualize the effect of each explanatory variable on the response variable [27,43]. Partial dependence plots are a useful tool for interpreting the results obtained from a random forest analysis and identifying patterns of the response variable based on each explanatory variable, including abrupt change points [27,44]. To date, however, few studies have applied the random forest algorithm and partial dependence plots to identify changes in the biological status of streams caused by changes in riparian land cover and their spatial patterns (including references of previous similar works).

We developed three random forest models for each biological indicator using 12 explanatory variables, including proportions of land cover (urban, agricultural, and forest areas) and land cover spatial patterns (LPI, PLAND, PD, ED for urban, agricultural, and forest patches). We employed the “randomForest” package for the statistical language R [45]. We set the number of trees (ntree) to 500 and the number of variables at each node of the tree (mtry) to the default value [27]. The value of mean decrease in accuracy (% IncMSE) was calculated to detect the importance of a variable; the greater the value of % IncMSE, the more important the value of the variable. We categorized 70% and 30% of the data as the training and testing datasets, respectively, to evaluate the performance of the models. The root mean square error (RMSE) and mean absolute error (MAE) were also used to assess the performance and measure the accuracy of the variables [46]. The lower the RMSE and MAE, the better the prediction ability of the model. Partial dependence plots were constructed using the “pdp’” package within R to explore the effects of all the variables in the model.

## 3. Results

### 3.1. Descriptive Statistics

The general results of the biological indicators, percentage of land cover types, and their spatial patterns are displayed in Table 3. The TDI, BMI, and FAI of the 770 monitoring sites exhibited mean values of 60.8, 66.8, and 63.0 (out of a maximum of 100). The TDI and FAI were categorized as “fair,” while the BMI was categorized as “good” (i.e., good quality biological communities) at most monitoring sites, based on the classifications of biological indicators in NAEMP. On the riparian buffer scale, the mean values of urban and agricultural land cover were 11.7% and 19.3%, respectively. Forest areas covered the highest percentage among the land cover types. The mean LPI and PLAND indices for forest areas were higher than those in urban areas and agricultural areas, while the mean PD and ED indices for forest areas were lower than those in urban and agricultural areas. In terms of the degree of fragmentation in the landscape, higher LPI and PLAND values and smaller PD and ED values indicated less fragmentation.

### 3.2. Random Forest Models for Biological Indicators

**Figure 2:**
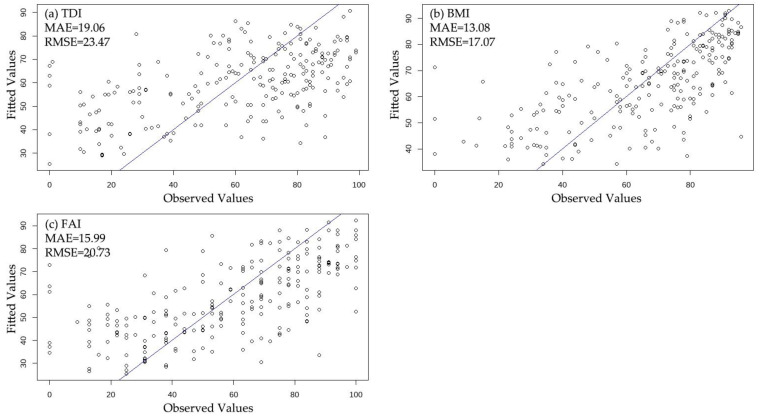
Comparison of random forest model performances for the estimation of biological indicators: (**a**) Trophic Diatom Index (TDI); (**b**) Benthic Macroinvertebrate Index (BMI); and (**c**) Fish Assessment Index (FAI). MAE, mean absolute error; RMSE, root mean square error.

Random forest models were developed for each biological indicator, and their performances were compared. The RMSE values for TDI, BMI, and FAI were 23.47, 17.07, and 20.73, respectively, while the MAE values were 19.06, 13.08, and 15.99, respectively (Figure 2). The results indicate that the random forest model exhibited a better performance with the Benthic Macroinvertebrate Index (BMI) than with the Trophic Diatom Index (TDI) and the Fish Assessment Index (FAI).

**Figure 3:**
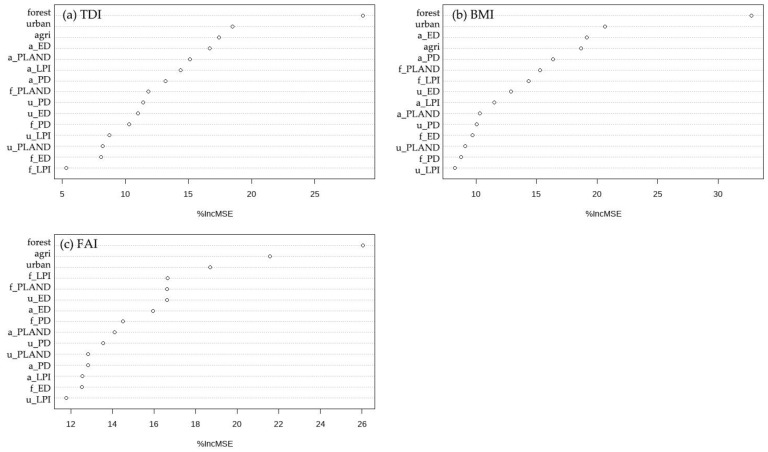
Relative influence of predictors based on the random forest models: (**a**) Trophic Diatom Index (TDI); (**b**) Benthic Macroinvertebrate Index (BMI); and (**c**) Fish Assessment Index (FAI). PD, patch density; ED, edge density; LPI, large patch index; PLAND, percentage of landscape. The vertical axis represents the number of variables, while the horizontal axis represents the relative influence of each variable.

The random forest algorithm ranked the relative importance of land cover characteristics in the riparian buffer zone for the TDI, BMI, and FAI (Figure 3). Higher values of percentage increase in the mean squared error (MSE) indicate higher importance. The results indicate that forest area in riparian buffer zones was the most important predictor for all biological indicators, whereas the following important factors varied across the biological indicators. The proportions of land cover in a riparian zone have a greater influence on aquatic ecosystems than spatial patterns in a riparian zone. The top five predictors of TDI were forest area (%), urban area (%), agricultural area (%), ED of agricultural area, and PLAND of agricultural area. The top five predictors of BMI were forest area (%), urban area (%), ED of agricultural area, agricultural area (%), and PD of agricultural area. These results indicate that TDI and BMI have common predictors and rankings. Additionally, the relative importance of % forest land cover in a riparian zone for TDI and BMI was significantly higher than that of any other variable. However, in terms of FAI, the proportions of all three riparian land cover types were ranked higher than other predictors.

### 3.3. The Partial Dependence Plots Analysis

**Figure 4:**
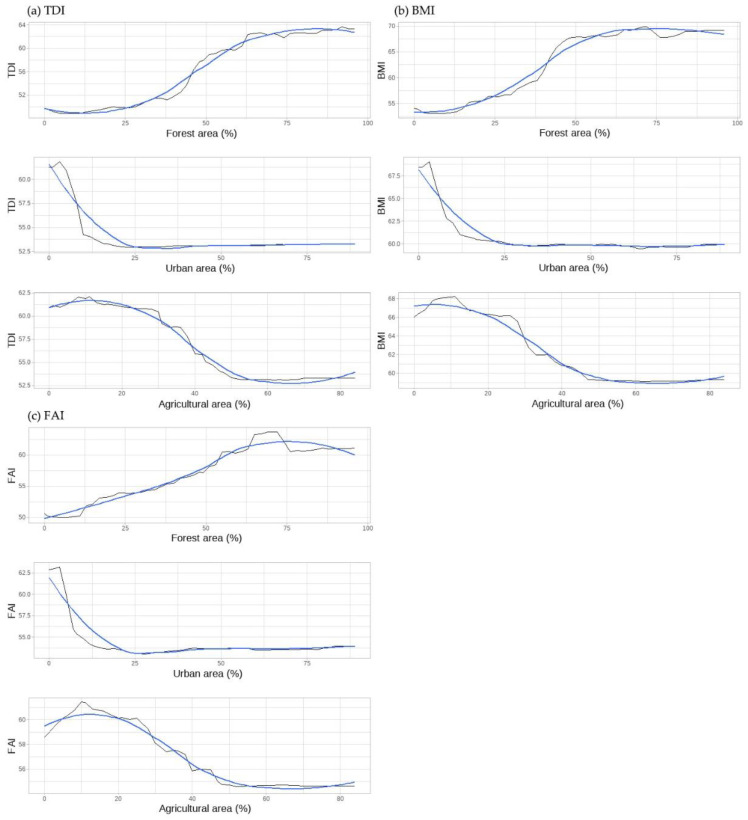
Partial dependence plots (black curves): (**a**) TDI; (**b**) BMI; and (**c**) FAI, based on random forests. Smooth curves are shown in blue.

We show PD plots for the proportions of forest, urban, and agricultural areas, which are identified as common important predictors of the biological indicators in the random forest models (Figure 4). The PD plots for biological indicators with proportions of riparian land cover types demonstrated similar patterns. According to the PD plots for the riparian forest area, when the forest area of a riparian zone was more than 60%, the effect on stream biological communities was not significantly changed. The biological values gradually increased until the proportion of forest area was approximately 60% of the riparian zone. The plot suggests that the greater the forest area in a riparian zone, the better the integrity of the biological communities of the stream. Conversely, the biological status of a stream gradually decreased until the percentage of urban area was 25% of the riparian zone. If the proportion of urban areas in a riparian zone exceeds 25%, the biological communities of streams remain in poor condition. The biological indicator values decrease until the agricultural area in a riparian zone reaches 60%. In particular, when the percentage of agricultural area is in the range of 20% to 40%, the biological status of the stream decreases abruptly.

## 4. Discussion

### 4.1. Influences of Riparian Land Cover Proportions and Patterns on the Biological Integrity of Streams

Many previous studies have researched the influence of land cover characteristics on biological communities in streams [47,48,49]. Riparian land cover proportions and patterns have generally been employed to assess the impacts of riparian land cover on the biological conditions in the stream [13,19]. The proportions of land cover have usually been identified as a better predictor of biological integrity in streams than land cover patterns, as presented in our study [50,51]. The results of this study indicate that the top five most influential land cover characteristics of a riparian zone on the three considered biological indicators included the proportion of urban, agricultural, and forest areas, although the importance values and rankings were slightly different. The results suggest that the percentage of certain land cover types in a riparian zone can better account for the variability in the biological status of streams than land cover patterns, such as LPI, PLAND, ED, and PD. The findings of this study illustrate that it may be more effective to improve biological conditions in streams by regulating the proportions of riparian land cover in our study area.

The results of this study suggest that the predictive capabilities of macroinvertebrate and fish models were better than those of diatoms in assessing the impacts of riparian land cover on the biological integrity of streams in the Han River basin. Diatoms are more sensitive to local perturbations than to watershed and riparian-scale land cover changes, likely due to their lower mobility, although they are sensitive to nutrients and organic pollution [48,52]. Conversely, macroinvertebrate and fish assemblages are more sensitive to watershed and riparian land cover, and can therefore be efficient indicators for assessing the influence of pollution originating from the surrounding area [52,53]. The results of this study suggest that more than one biological organism should be considered when assessing the impact of environmental variables, as suggested in previous literature [52,53].

The influence of land cover characteristics on stream-based biological communities differs depending on the aquatic organisms under consideration [53,54,55]. Each biological assemblage responds differently to riparian land cover types, as they exhibit diverse behavioral traits, life histories, and sensitivities to stressors [52]. As indicated by the variable importance plot in our study, the strength of riparian land cover as an indicator of overall fish assemblage integrity was higher than that of diatoms and macroinvertebrates. However, these results varied across the spatial scales, study areas, and biological metrics variously used in the existing literature [53,54,55]. For example, Flinders et al. [54] showed that fish indices can be the better predictor of land cover than macroinvertebrates. Walters et al. [53], on the other hand, found that macroinvertebrates were the better predictor of land cover. Our findings are consistent with those of previous studies in that we identify fish indices as being useful in assessing riparian land cover as fish are more mobile than most other riverine organisms, and therefore exhibit sensitivities to change on a broader geographical scale [54,55]. Conversely, macroinvertebrate community structure was more sensitive to local-scale stressors such as nutrient concentration, sedimentation, and substrate coarseness [47,54].

Among the proportions of riparian land cover, riparian forest areas had the greatest effect on the three considered biological communities, followed by riparian urban areas and agricultural areas. These results are consistent with those of many studies that have shown that riparian forests play an important role in sustaining biological integrity in streams [13,15,19]. Carlisle et al. [44] concluded that riparian forests are a more important predictor of biological integrity in streams than riparian urban and agricultural areas. Riparian vegetation has been shown to have various benefits for stream conditions, such as intercepting rainfall, slowing surface runoff speed, capturing pollutants and sediments, and providing habitats for aquatic organisms [56,57,58]. In particular, riparian forest cover has been shown to play an important role in mediating the negative impacts of land cover on streams [59]. Even if the watershed is dominated by agricultural areas, riparian forests can effectively mitigate the negative impacts of agricultural land cover [60]. In an applied management context, it is important to protect the riparian forest from fragmentation and changes to other land cover types.

Urban and agricultural areas in the watershed and riparian zone negatively influence the quality of aquatic organism habitats, affecting their structure and composition [61]. Urban and agricultural runoff mainly contributed to increasing nutrient concentrations and sediment inputs, reducing stream substrate coarseness, and driving a shift within the local biological community from sensitive species to more pollution-tolerant species [47,48]. Riparian forests play critical roles in maintaining the biological integrity of streams as they primarily determine instream habitat quality and pollutant inputs [47,49], as described in the paragraph above. Moreover, several studies reported that even in headwater streams, where most of the watershed land cover consists of vegetation, riparian deforestation or impairment has a strong impact on the instream habitat quality of biological communities. We therefore conclude that riparian conditions are the most important factors in maintaining the biological integrity of streams [62,63].

### 4.2. Threshold Effects of Land Cover Characteristics on the Biological Integrity of Streams

Many previous studies have shown that the responses of stream conditions to land cover characteristics are nonlinear and that abrupt change points exist, which are called thresholds [16,28,29,30,64]. Human actions such as causing changes in land cover exhibit thresholds that suddenly change the stable state of ecosystems, while ecosystems require an enormous amount of time and effort to recover. Therefore, it is important to predict such thresholds and identify a precise approach. In this study, we found that there are some important thresholds, where the effects of land cover on stream biological status abruptly change, based on the partial dependence plots from the random forest models. The proportion of forested area should be more than 60% to assist in setting targets for environmental conservation. The results suggest that if more than 60% of green space in a riparian zone is covered by forests, the biological conditions in streams can be maintained at good quality. Clément et al. [17] have shown that securing forest cover of more than 50% can mediate the negative effects of agricultural areas on Canadian streams. In the present study, biological conditions in streams continued to decrease until the percentage of urban area in the riparian area was approximately 25%. Similarly, King et al. [65] concluded that significant changes in benthic macroinvertebrates occurred at a threshold of 20–30% developed area. These results suggest that when developing a watershed, it is critical to limit the proportion of urban area to within 25%, in order to minimize the impact on the biological status of streams, especially in riparian zones. The partial dependence plots also showed that the values of biological indicators abruptly decreased when the agricultural area in a riparian zone was between 20% and 40%. Utz et al. [31] concluded that the threshold of agricultural land cover is higher than that of urban cover, which appears to be less damaging to aquatic macroinvertebrates. In summary, retaining more than 60% of the vegetated area in a riparian buffer, and not exceeding 25% of urban area and 20% of agricultural area are important criteria for maintaining the biological conditions in streams. Identifying abrupt changes in stream biological status caused by changes in land cover can assist managers and policymakers in establishing ecosystem conservation or restoration goals based on objective scientific criteria. In particular, our results can be applied to the planning and design of riparian buffer zones. However, more threshold analyses on the relationships between riparian land cover characteristics and stream biological status should be conducted, as there are some limitations; for example, the threshold effect may vary across the region, spatial scale, and stream condition indicators.

## 5. Conclusions

Our results demonstrate that the proportions of riparian land cover types offer a more powerful factor of stream-based biological community integrity than riparian land cover patterns. Conversely, when evaluating the impacts of land use, models informed by macroinvertebrate and fish have been shown to have greater predictive power than the diatom-informed model. Studies have also shown that fish could be considered the most efficient indicator when evaluating riparian land cover impacts. Our results clearly indicate that riparian forests play a significant role in determining the biological integrity of streams. We observe thresholds in the relationships between riparian land cover characteristics and stream-based biological indicators. Specifically, thresholds in riparian urban areas were lower than those in riparian agricultural areas, and more riparian forests had a positive influence on the biological indicators. Our results suggest that the proportions of riparian land cover should be considered as a quantitative criterion for riparian zone management and restoration. In particular, threshold analysis provides a quantitative standard for riparian land cover planning. Our results also imply that an ideal approach could involve restoring riparian forest to mitigate the impacts of urban and agricultural land use and protect the biological integrity of stream ecosystems. The results of this study provide essential insights which may help inform decision-making processes where riparian land cover planning, management, and restoration is concerned.

## Figures and Tables

**Table 1:** Equations for computing biological indicators, from the Korean Ministry of Environment (MOE) [38].

Biological Indicators	Equations
Trophic Diatom Index (TDI)	TDI = 100 − {(WMS × 25) − 25} WMS: weighted mean sensitivity $WMS=\sum A_{j}\cdot S_{j}\cdot \frac{V_{j}}{\sum A_{j}\cdot V_{j}}$ where, *j* = species *A_j_* = abundance (proportion) of species *j* in the sample (%) *S_j_* = pollution sensitivity (1 ≤ *S* ≤ 5) of species *j* *V_j_* = indicator value (1 ≤ *V* ≤ 3)
Benthic Macroinvertebrate Index (BMI)	$\mathrm{BMI}=\left\{4-\overset{n}{\underset{j=1}{\sum }}S_{j}H_{j}G_{j}/\overset{n}{\underset{j=1}{\sum }}H_{j}G_{j}\right\}\times 25$ where, *j* = number assigned to species *n* = number of species *S_j_* = unit saprobic value of species *j* *H_j_* = frequency of species *j* *G_j_* = indicators weight value of species *j*
Fish Assessment Index (FAI)	FAI = sum of 8 metrics. Metric 1 (M1): number of Korean native species Metric 2 (M2): number of rifle benthic species Metric 3 (M3): number of sensitive species Metric 4 (M4): percentage of tolerant species Metric 5 (M5): percentage of omnivores Metric 6 (M6): percentage of insectivores Metric 7 (M7): the amount of collection native species Metric 8 (M8): percentage of fish abnormalities

**Table 2:** Landscape metrics used to quantify land cover spatial patterns in this study.

Metrics	Description
Large patch index (LPI)	The area of the largest patch divided by the total land cover area.
Percentage of landscape (PLAND)	The sum of the areas of all patches divided by the total land cover area.
Patch density (PD)	The number of patches divided by the total land cover area.
Edge density (ED)	The sum of the lengths of the patches divided by the total land cover area.

Four metrics for urban, agricultural, and forest land cover (patches) were calculated individually.

**Table 3:** Descriptive statistics of biological indicators, percentage of land cover types, and the spatial patterns in the riparian buffer zones.

Classification	Variables	Mean	S.D.	Min	Max
Biological indicators	TDI (0–100)	60.8	26.7	0.0	99.0
	BMI (0–100)	66.8	23.3	0.0	96.0
	FAI (0–100)	63.0	26.1	0.0	100.0
Proportions of land cover	Urban area (%)	11.7	14.7	0.0	89.0
	Agricultural area (%)	19.3	16.2	0.0	84.0
	Forest area (%)	50.0	25.8	0.0	96.0
Land cover spatial patterns	Urban_LPI	12.3	18.7	0.0	92.0
	Urban_PLAND	21.6	24.9	0.0	92.0
	Urban_PD	52.4	36.9	0.0	224.0
	Urban_ED	125.9	76.2	6.0	486.0
	Agricultural_LPI	8.2	13.4	0.0	95.0
	Agricultural_PLAND	23.3	21.3	0.0	95.0
	Agricultural_PD	22.8	21.3	0.0	155.0
	Agricultural_ED	112.4	65.2	1.0	395.0
	Forest_LPI	15.4	18.6	0.0	96.0
	Forest_PLAND	34.0	27.4	0.0	96.0
	Forest_PD	18.8	28.3	0.0	168.0
	Forest_ED	86.7	56.5	0.0	415.0

n = 770; S.D., standard deviation; Min, minimum; Max, maximum; TDI, Trophic Diatom Index; BMI, Benthic Macroinvertebrate Index; FAI, Fish Assessment Index; LPI, large patch index; PLAND, percentage of landscape; PD, patch density; ED, edge density.
